# Endoscopic visualization of CT-confirmed accessory maxillary ostium: a retrospective study

**DOI:** 10.3389/fsurg.2026.1882855

**Published:** 2026-07-31

**Authors:** Majid Bani-Ata, Mohammed Qormat, Mohammed Baker, Leen Jaber, Mais Haitham, Osama Bdarnh, Ahmad Bani-Ata, Fayez M. Etoom, Atef Hulliel

**Affiliations:** 1 Division of Otolaryngology, Department of Special Surgery, Faculty of Medicine, Jordan University of Science and Technology, Irbid, Jordan; 2 Faculty of Medicine, Jordan University of Science and Technology, Irbid, Jordan; 3 Faculty of Science, Philadelphia University, Jerash, Jordan

**Keywords:** accessory maxillary ostium, CT scan, fiberoptic endoscopy, nasal septum, polyp

## Abstract

**Background:**

The accessory maxillary ostium (AMO) is a common anatomical variant of the lateral nasal wall and has been implicated in impaired maxillary sinus drainage, mucus recirculation, and chronic sinus pathology. Although AMO can be identified using both radiologic and endoscopic techniques, the endoscopic visualization rate of CT-documented AMO has not been well characterized. This study aimed to evaluate the endoscopic visualization rate of AMO using FFE in patients with CT-documented AMO.

**Method:**

This retrospective cohort study was conducted at a tertiary referral center between January 2023 and December 2024. Patients with CT-confirmed AMO who subsequently underwent FFE were included. Patients with a history of prior sinus surgery were excluded. Demographic data, CT findings, and endoscopic visualization of AMO were collected from electronic medical records. Descriptive statistics were used to summarize the data, and categorical variables were analyzed using Chi-square or Fisher's exact tests as appropriate.

**Results:**

A total of 274 patients were included, with a mean age of 34.4 ± 14.1 years; 51.8% were male. While AMO was confirmed on CT imaging in all participants by inclusion criteria, FFE visualized AMO in only 26% of cases (71/274), indicating a low endoscopic visualization rate among patients with CT-confirmed AMO. Maxillary sinus opacification was detected on CT in 49.6% of patients, while nasal polyps were present in 34.3%. No significant gender differences were observed in the prevalence of maxillary opacification or nasal polyps.

**Conclusion:**

Flexible fiberoptic endoscopy demonstrated limited ability to visualize accessory maxillary ostium in patients with CT-documented AMO. While FFE remains useful for functional and mucosal assessment, CT imaging appeared to useful for anatomical identification of AMO and may be considered for comprehensive evaluation and surgical planning.

## 1 Introduction

Anatomically, the accessory maxillary ostium (AMO) is most frequently located in the membranous region on the lateral wall of the nasal cavity, between the uncinate process and the inferior turbinate (1). This area is classically divided into anterior and posterior segments by the ethmoid projection of the inferior concha (1). In most individuals, the natural maxillary ostium (NMO) aligns with the anterior portion of the posterior fontanelle, forming the primary drainage pathway of the sinus (2). The presence of an accessory ostium in this location may disturb that delicate drainage, contributing to mucus recirculation, impaired mucociliary clearance, and chronic mucus retention, even in the absence of obvious infection (1–3).

The AMO represents a significant anatomical variation, with prevalence reported between 18% and 30% of the general population (3). Imaging studies indicate that AMO appears unilaterally in nearly one-third of individuals on each side and bilaterally in close to one-fifth of the population (4). Maxillary sinusitis is frequently observed in these individuals, affecting both sides at comparable rates, with a substantial pro-portion demonstrating bilateral involvement (5, 6).

Individuals with AMO also show a higher likelihood of presenting with mucus retention cysts (approximately 48.6%) and mucosal thickening (about 43%), while the prevalence of maxillary sinus (MS) inflammation may reach nearly 29.1% in this group (7). Cone-Beam Computed Tomography (CBCT) studies have reported even higher prevalence rates, with AMO detected in up to 65% of examined maxillary sinuses, reflecting differences in imaging protocols and patient populations (3, 7). Moreover, CBCT studies have demonstrated a significant anatomical association between AMO and Haller cells, with 39.5% of right-sided and 34.5% of left-sided sinuses exhibiting AMO, often accompanied by localized mucosal thickening in cases of bilateral AMO (8).

AMO has been associated with variations in adjacent structures, including Haller cells, concha bullosa, and nasal septal deviations, highlighting the interplay between sinus anatomy and pathological changes (5). These associations suggest that AMO may influence both structural and functional aspects of MS, potentially predisposing to chronic mucus retention and cystic lesion formation even in the absence of overt infection (5, 9).

AMO can be identified using both radiologic and endoscopic approaches, which complement each other. Advanced imaging techniques, such as computed tomography (CT) and CBCT, provide detailed visualization of the ostium's precise location, dimensions, and morphology, as well as its relationship with adjacent bony structures and anatomical variants like Haller cells or concha bullosa (3, 4, 10). These modalities are particularly valuable in detecting AMO that may be partially obscured by mucosal tissue or hidden posteriorly. Nasal endoscopy, including flexible fiberoptic endoscopy (FFE), allows functional assessment, such as observation of mucus flow and ostial patency, though it may be limited in visualizing ostia situated behind the uncinate process. FFE can be performed in routine clinical settings, making it practical and patient-friendly, and is particularly valuable for assessing mucosal status and drainage patterns that are not visible on imaging alone. However, endoscopic detection is limited by anatomical and operator-dependent factors. Combining imaging and endoscopy improves detection accuracy and directing surgical efforts, particularly for functional endoscopic sinus surgery (FESS) (11, 12).

Therefore, this study aimed to evaluate the endoscopic visualization rate of accessory maxillary ostium using flexible fiberoptic endoscopy among patients with CT-confirmed AMO and to identify factors associated with successful endoscopic visualization.

## 2 Methods and materials

### 2.1 Study design

This was a retrospective cohort study conducted at King Abdullah University Hospital (Irbid, Jordan) between January 2023 and December 2024, aiming to evaluate the endoscopic visualization of accessory maxillary ostium using flexible fiberoptic endoscopy in patients.

### 2.2 Participants and eligibility criteria

We included patients in whom accessory maxillary ostium was identified using CT scan and who subsequently underwent flexible fiberoptic nasal endoscopy at our center. Patients with a previous history of sinus surgery were excluded to avoid anatomical distortion that could bias the results.

### 2.3 Instruments and data collection

Patients' data were collected through a review of electronic medical records at our center. Each patient was assigned a unique code to ensure data anonymization. Variables were collected using a predesigned Excel spreadsheet and included age, sex, history of sinus surgery, history of nasal polyps, endoscopic presence of accessory maxillary ostium using flexible fiberoptic endoscopy, and radiological findings related to the maxillary sinus when available. All collected data were stored on a secure personal computer prior to analysis.

### 2.4 Statistical analysis

All data analyses were performed using the IBM Statistical Package for the Social Sciences (SPSS) software for Windows, version 26.0. Descriptive measures included mean ± standard deviations for continuous data if the normality assumption was not violated, according to the Shapiro–Wilk test, and median with first and third quartiles (Q1-Q3) if the assumption was violated. Categorical data were presented by frequencies and percentages (%). The Chi-square test was used for categorical variables, and Fisher's exact test was applied when expected frequencies were below the acceptable threshold. Because maxillary sinus opacification and nasal polyps each completely separated the outcome, a Firth penalized-likelihood logistic regression was used to obtain finite, bias-reduced estimates. Given the complete collinearity between the two findings (all patients with polyps also had opacification), they were combined into a single obstructing-pathology term (opacification and/or nasal polyps), with age and sex retained as covariates. Penalized profile-likelihood 95% confidence intervals and *p*-values are reported. Analyses were performed in R using the logistf package.

### 2.5 CT imaging protocol

Non-contrast CT imaging of the paranasal sinuses was performed using a multidetector CT scanner at the Department of Radiology. All scans were acquired in the supine position without gantry tilt, covering from the hard palate to the superior margin of the frontal sinuses. Acquisition parameters included a slice thickness of 0.625–1 mm, tube voltage of 120 kVp, and tube current of approximately 100–250 mAs. Images were reconstructed in the axial plane and reformatted in the coronal and sagittal planes using both bone (window width: 2,700 HU, window level: 700 HU) and soft tissue (window width: 400 HU, window level: 40 HU) kernel settings. The accessory maxillary ostium was defined on CT as a bony defect in the membranous portion of the lateral nasal wall (posterior or anterior fontanelle), distinct from and not contiguous with the natural maxillary ostium, demonstrating visible communication between the nasal cavity and the maxillary sinus on at least two consecutive coronal slices. All CT scans were reviewed by a board-certified radiologist. Additional CT findings, including maxillary sinus opacification and the presence of nasal polyps, were documented when present. The principal investigator was blinded to the CT scan results.

### 2.6 Flexible fiberoptic endoscopy protocol

Flexible fiberoptic endoscopy was performed in the outpatient clinic setting using a flexible nasopharyngoscope. Prior to the procedure, topical nasal decongestion and local anesthesia were applied. The endoscope was advanced through the nasal cavity to systematically examine the inferior meatus, middle meatus, and nasopharynx. The middle meatus was inspected for the presence of an accessory maxillary ostium, defined endoscopically as a visible opening in the posterior fontanelle region of the lateral nasal wall, separate from the natural maxillary ostium and the ethmoidal infundibulum. The presence or absence of AMO visualization, along with any associated mucosal findings, was documented in the electronic medical record. All endoscopic examinations were performed by otolaryngologists at our center; however, the specific operator and level of experience were not systematically recorded due to the retrospective nature of the study. Therefore, their potential effect on visualization rates could not be evaluated and may have contributed to the observed variability.

### 2.7 Ethical consideration

This study was conducted in accordance with the principles of the Declaration of Helsinki and institutional review board approval was obtained.

## 3 Results

### 3.1 Participants' characteristics

A total of 274 participants met the inclusion criteria. 51.8% were males (*n* = 142). The mean age was 34.4 years (SD ±14.1). Table 1 describes the participants' demographics.

**Table 1:** Patient demographics.

Demographics	*N* (%) or Mean (SD)
Total Patients	274
Mean Age	34.4 Years (SD ± 14.1)
Age Range	13–83 Years
Male	142 (51.8%)
Female	132 (48.2%)

### 3.2 Detection rate of accessory maxillary ostium

**Figure 1:**
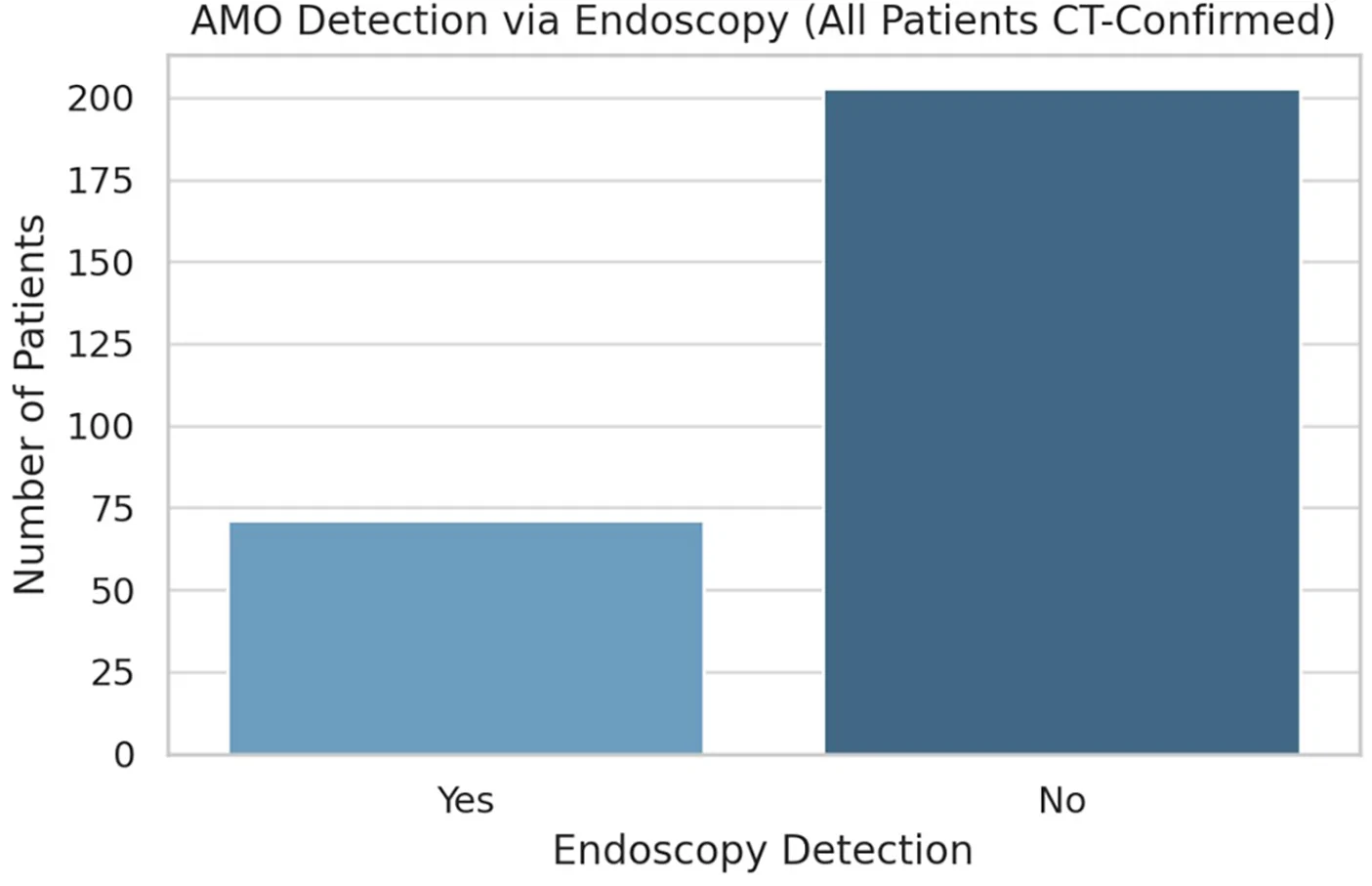
Detection of accessory maxillary ostium (AMO) via endoscopy in patients with CT-confirmed AMO.

**Figure 2:**
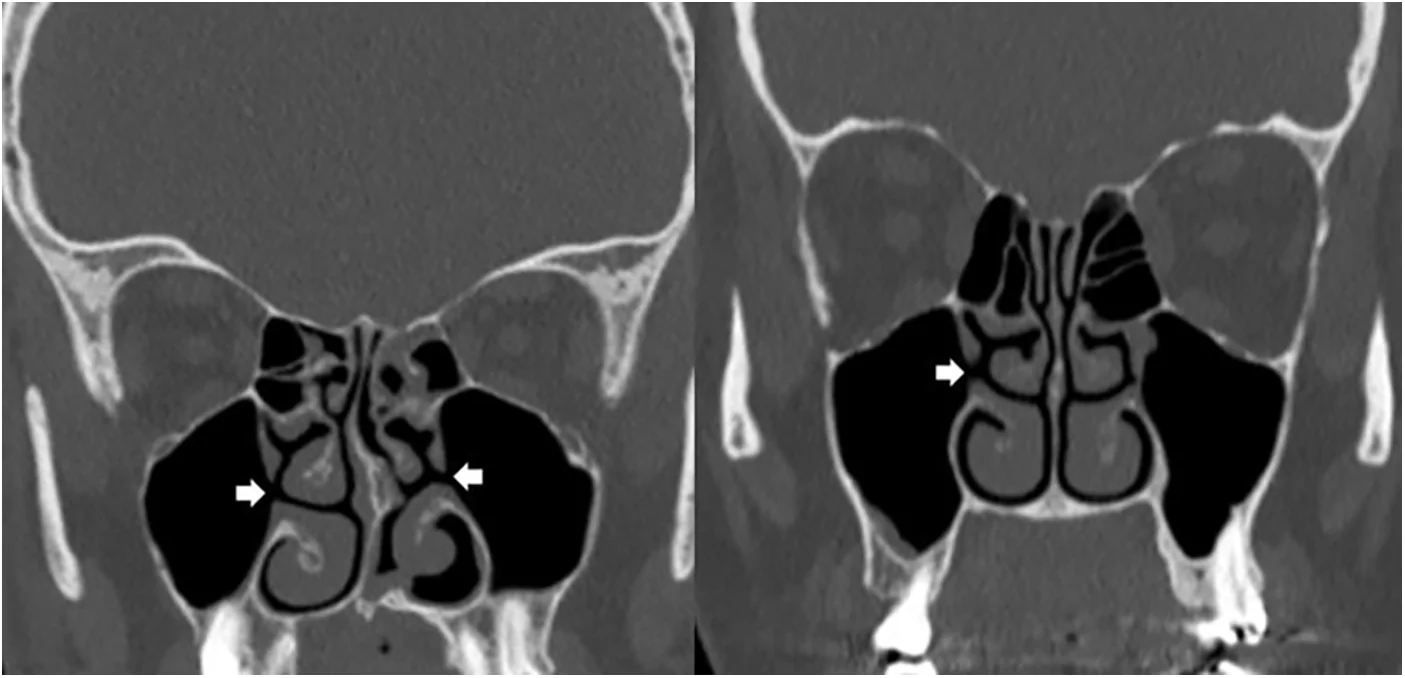
Coronal non-contrast CT images of the paranasal sinuses demonstrating accessory maxillary ostia (white arrows).

**Figure 3:**
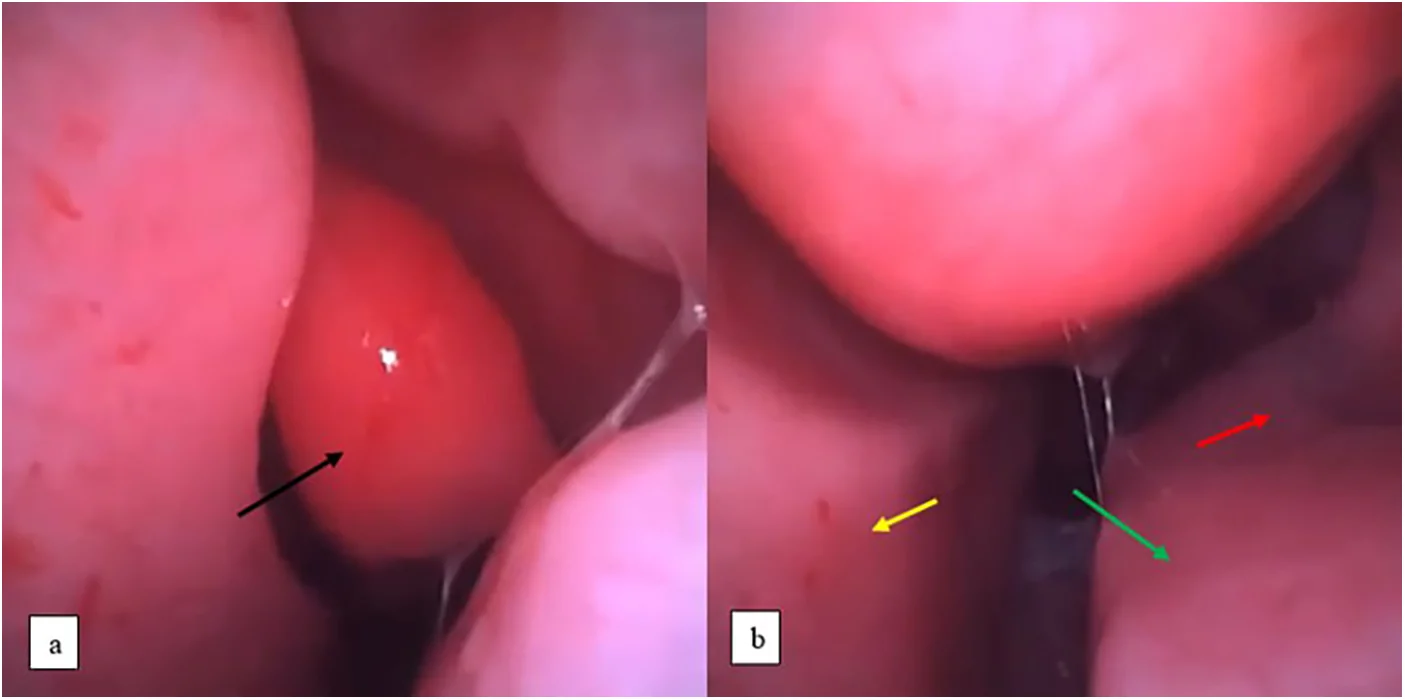
Flexible fiberoptic endoscopic view of the middle meatus in a patient with CT-confirmed accessory maxillary ostium (AMO). **(a)** Endoscopic view of the middle meatus; the black arrow indicates the middle turbinate. **(b)** The same field with anatomical landmarks labelled: yellow arrow, nasal septum; red arrow, middle meatus; green arrow, inferior turbinate.

According to the inclusion criteria, accessory maxillary ostium was documented radiologically by CT imaging in all included cases. Flexible fiberoptic endoscopy identified accessory maxillary ostium in 26% of participants (71/274) (Figure 1). Representative imaging from patients with CT-confirmed AMO that was not visualized endoscopically is shown in Figures 2 and 3, illustrating the coronal CT demonstration of the accessory ostium (Figure 2**)** and the corresponding endoscopic findings (Figure 3).

### 3.3 CT detection of maxillary opacification

We were able to identify maxillary opacification using CT in 136 out of the original 274 participants (49.6%). Table 2 shows the cross tabulations of maxillary opacifications and gender with a slightly higher prevalence in male participants (52.1%) compared to (47.0%) in females. Fisher's exact test showed no significant difference between two genders in the presence of maxillary opacification (*p* = 0.4).

**Table 2:** CT detected maxillary opacification by gender.

Gender	Not Detected	Detected	Total
Female	70	62 (47.0%)	132
Male	68	74 (52.1%)	142
Total	138	136 (49.6%)	274

### 3.4 CT detection of nasal polyps

Nasal polyps were detected in 94 participants (34.3%) using CT. Table 3 illustrates the prevalence by gender, showing a slightly higher prevalence in males (37.3%) compared with females (31.0%). Fisher's exact test showed no significant difference between the two genders in the presence of nasal polyps (*p* = 0.31).

**Table 3:** CT detected nasal polyps by gender.

Gender	Not Detected	Detected	Total
Female	91	41 (31.0%)	132
Male	89	53 (37.3%)	142
Total	180	94 (34.3%)	274

### 3.5 Comparison of FFE-positive and FFE-negative patients

A comparison of clinical and demographic characteristics between patients in whom FFE visualized AMO (FFE+, *n* = 71) and those in whom it did not (FFE-, *n* = 203) is presented in Table 4. There was no significant difference in mean age between the two groups (34.1 ± 15.9 vs. 34.5 ± 13.4 years, *p* = 0.397). Males comprised 43.7% of the FFE + group and 54.7% of the FFE- group; this difference was not statistically significant (*p* = 0.129). Notably, none of the 71 patients in whom FFE visualized the AMO had CT-detected maxillary opacification (0.0% vs. 67.0%, *p* < 0.001) or nasal polyps (0.0% vs. 46.3%, *p* < 0.001).

**Table 4:** Comparison of demographic and clinical characteristics between patients in whom FFE visualized AMO (FFE+) and those in whom it did not (FFE−).

Variable	FFE+ (*n* = 71)	FFE− (*n* = 203)	*p*-value
Age (mean ± SD)	34.1 ± 15.9	34.5 ± 13.4	0.397ᵃ
Male, *n* (%)	31 (43.7%)	111 (54.7%)	0.129ᵇ
CT Maxillary Opacification, *n* (%)	0 (0.0%)	136 (67.0%)	<0.001ᵇ
Nasal Polyps, *n* (%)	0 (0.0%)	94 (46.3%)	<0.001ᵇ

ᵃ Mann–Whitney *U*-test.

ᵇ Fisher's exact test.

### 3.6 FFE detection rate by Sinus pathology

Stratification by the presence of sinus pathology revealed marked differences in FFE detection rates Table 5. Among patients without maxillary opacification (*n* = 138), FFE detected AMO in 51.4% (71/138) of cases. In contrast, FFE failed to visualize AMO in any of the 136 patients with CT-confirmed maxillary opacification (*p* < 0.001). A similar pattern was observed for nasal polyps: FFE detected AMO in 39.4% (71/180) of patients without polyps, but in none of the 94 patients with polyps (*p* < 0.001). Among the 138 patients with neither opacification nor polyps, the FFE detection rate was 51.4%.

**Table 5:** Endoscopic detection rate of AMO stratified by presence of CT-detected maxillary opacification and nasal polyps.

Subgroup	*n*	FFE+ *n* (%)	FFE− *n* (%)	*p*-value
Without maxillary opacification	138	71 (51.4%)	67 (48.6%)	<0.001ᵃ
With maxillary opacification	136	0 (0.0%)	136 (100%)	
Without nasal polyps	180	71 (39.4%)	109 (60.6%)	<0.001ᵃ
With nasal polyps	94	0 (0.0%)	94 (100%)	
Neither opacification nor polyps	138	71 (51.4%)	67 (48.6%)	–

ᵃ Chi-square test comparing detection rates between subgroups.

### 3.7 Detection rate by Age group and gender

The FFE detection rate did not differ significantly across age groups (*χ*²=3.913, df = 4, *p* = 0.418; Table 6), although the youngest group (≤20 years) showed a numerically higher detection rate (35.8%) compared with other age groups (20.9–26.8%). Females showed a non-significantly higher FFE detection rate compared with males (30.3% vs. 21.8%; OR = 0.64, 95% CI: 0.37–1.11, *p* = 0.129). Logistic regression analysis with age and gender as predictors confirmed that neither variable was significantly associated with FFE detection of AMO (gender: OR = 0.64, *p* = 0.11; age: OR = 1.00, *p* = 0.87).

**Table 6:** FFE detection rate of AMO by age group.

Age Group	Total (*n*)	FFE+ *n* (%)	FFE− *n* (%)	*p*-value
≤20 years	53	19 (35.8%)	34 (64.2%)	0.418ᵃ
21–30 years	69	17 (24.6%)	52 (75.4%)	
31–40 years	67	14 (20.9%)	53 (79.1%)	
41–50 years	44	10 (22.7%)	34 (77.3%)	
>50 years	41	11 (26.8%)	30 (73.2%)	

ᵃ Chi-square test.

A Firth penalized-likelihood logistic regression using a combined obstructing-pathology term (opacification and/or polyps), adjusted for age and sex, confirmed that obstructing sinus pathology was strongly and independently associated with failure to visualize AMO endoscopically (adjusted OR 0.003, 95% CI <0.001–0.025, *p* < 0.001).

## 4 Discussion

The accessory maxillary ostium (AMO) represents a common anatomical variant of the lateral nasal wall, the clinical significance of which remains a subject of ongoing debate in rhinology (1, 2). The present study aimed to evaluate the detection rate of AMO using flexible fiberoptic endoscopy (FFE) in a cohort of patients where the presence of AMO was confirmed radiologically by computed tomography (CT). Our primary finding is that FFE successfully identified the AMO in 26% (71/274) of the radiologically positive cases. More importantly, our subgroup analysis revealed a complete dichotomy: all 71 patients in whom FFE visualized AMO had neither maxillary opacification nor nasal polyps, while none of the patients with these pathological findings had endoscopically visible AMO (*p* < 0.001 for both). This observation highlights the inherent limitations of FFE as a standalone diagnostic tool for AMO, while simultaneously underscoring its role in the functional assessment of the middle meatus (12, 13).

The reported prevalence of AMO in the general population varies widely, ranging from 20% to over 65%, depending on the diagnostic modality and the population studied (7, 14). Yenigun et al. reported that AMO was present in 19.1% (72/377) of patients, and that with its presence, the probability of encountering a mucus retention cyst was approximately 48.6%, mucosal thickening was approximately 43.0%, and maxillary sinusitis was approximately 29.1% (3). Shetty et al. found that sinuses with AMOs showed a significantly greater frequency of mucosal thickening (*p* = 0.001), and that AMOs with larger maximal length were associated with higher degrees of mucosal thickening (15). These findings, together with our high prevalence of maxillary opacification (49.6%) and nasal polyps (34.3%) in our AMO-positive cohort, align with the established literature linking AMO to sinus pathology (6).

The most significant finding of the present study is the complete absence of endoscopic AMO visualization in patients with maxillary sinus opacification or nasal polyps. Among patients without opacification, FFE detected AMO in 51.4% of cases, a rate substantially higher than the overall 26% and more consistent with what might be expected from endoscopic examination of a patent middle meatus.

Rudbarizade et al. reported that in their CBCT study, the presence or absence of AMO could not be determined in 14.63% of all sinuses due to complete sinus opacification or peripheral increase in sinus mucosal thickness, and that in sinuses with normal mucosal status (mucosal thickness less than 2 mm), the number of sinuses with AMO was significantly higher than in sinuses with abnormal mucosal status (*p* < 0.001) (16).

Several factors contribute to the lower endoscopic detection rate. The AMO is typically located in the posterior fontanelle, a region that can be challenging to visualize endoscopically due to the overlying uncinate process, the presence of mucosal edema, or anatomical crowding within the middle meatus (17, 18). Furthermore, the size and patency of the AMO are dynamic, and a small, slit-like ostium may be obscured by normal mucosal folds or subtle inflammation, even if it is structurally present (9). In patients with maxillary opacification, the inflammatory process itself, characterized by edematous thickening of the sinus mucosa and accumulation of mucopurulent secretions, likely obliterates the endoscopic visibility of the AMO by engulfing the posterior fontanelle region. Similarly, nasal polyps arising from or extending into the middle meatus physically obstruct the endoscopic field, rendering the fontanelle and any ostia within it inaccessible to FFE visualization.

Lohiya et al. compared diagnostic nasal endoscopy (DNE) and CT in diagnosing chronic rhinosinusitis, reporting that endoscopy had a sensitivity of 88.04% and specificity of 28.57% when compared to CT as the gold standard, concluding that there was no significant difference in diagnosing CRS by either modality (19). However, their study focused on overall CRS diagnosis, not on the identification of specific anatomical variants such as AMO. Krishniya similarly reported that the sensitivity of endoscopy was 93.18% and specificity was 83.33% when compared with CT for CRS diagnosis (20). Our findings suggest that while endoscopy and CT may have comparable overall diagnostic performance for CRS, the two modalities differ substantially in their ability to identify specific anatomical features, particularly in the presence of obstructing pathology.

The clinical relevance of AMO is primarily linked to the phenomenon of mucus recirculation, a critical concept in the pathogenesis of chronic maxillary sinusitis (16). Gutman described mucous recirculation as a process in which secretions exiting through the natural maxillary ostium re-enter the sinus via a surgically created or accessory ostium. They noted that this phenomenon may contribute to persistent sinus infection (21). Chung et al. analyzed CT findings of mucous recirculation between the natural and accessory ostia, showing that the primary mucous recirculation is demonstrated as a ring structure in coronal CT scans (22). Bani-Ata et al. reported in their study of 928 patients that a right accessory maxillary ostium was detected in 29.5% and bilateral ostia were found in 172 patients, with the presence of right maxillary sinusitis being significantly associated with the presence of a right accessory maxillary ostium (4). Orhan Soylemez found that in the AMO-positive group, sinusitis, mucosal thickening, and primary ostium obstruction were significantly more common than in the AMO-negative group (*p* < 0.00001) (9). Çapar reported that sex and the presence of an AMO emerged as independent predictors of maxillary sinus pathologies (8).

The lack of a significant gender difference in the prevalence of opacification and polyps in our cohort is consistent with findings from other large-scale studies. Bani-Ata et al. found no gender differences in the prevalence of accessory maxillary ostia overall (4). Kashi et al. reported that the presence of AMO had no significant correlation with maxillary sinusitis (*p* = 0.104) (23), and Rudbarizade et al. noted that age was not an influencing factor in the presence of AMO (*p* = 0.361) (16).

The low FFE detection rate in our study, particularly the detection rate in patients with sinus pathology, reinforces the necessity of preoperative CT imaging for comprehensive anatomical mapping in the context of Functional Endoscopic Sinus Surgery (8).

CT provides a comprehensive assessment of maxillary sinus anatomy, including areas that may be obscured on endoscopy, facilitating anatomical identification of AMO. This improved detection is clinically relevant, as unrecognized AMO may contribute to impaired sinus drainage and persistent disease through mucus recirculation. Therefore, CT appeared more suitable for anatomical documentation of AMO, particularly when precise anatomical delineation is required for clinical management or surgical planning.

In the present study, CT imaging served as the radiologic comparator because it provides high-resolution visualization of sinonasal anatomy, including the posterior fontanelle region. Unlike endoscopy, CT allows assessment of anatomical structures regardless of mucosal edema, polyposis, or obstruction of the endoscopic field. Therefore, the findings should be interpreted as comparisons against CT-documented AMO rather than true diagnostic accuracy measures.

This study has several limitations. The retrospective design limits prospective follow-up and faces challenges related to missing data due to variations in documentation. Our study included patients who already had AMO identified via CT, and therefore evaluates the endoscopic visualization of CT-documented AMO in a preselected AMO-positive cohort rather than diagnostic accuracy in a general population.

This design inherently precludes the calculation of sensitivity and specificity for FFE. Additionally, limitations of FFE such as the non-standardization of the procedure and variability of clinicians performing the examination may affect the efficiency of FFE in detecting AMO, likely resulting in an underestimation of the true detection rate. Moreover, the potential for classification bias should be acknowledged, as inflammatory sinonasal disease, mucosal edema, and anatomical distortion may substantially obscure the posterior fontanelle region, thereby limiting endoscopic identification of AMO despite its radiological confirmation on CT imaging.

Furthermore, the presence of opacification and polyps as complete barriers to FFE detection may reflect both a true obstructive effect and a selection bias, as patients with more severe sinus disease may have a different AMO profile. Future prospective studies with standardized FFE protocols, documented operator experience, and systematic recording of AMO morphology on CT should be conducted to further clarify the determinants of endoscopic AMO detection. Another limitation is that all CT examinations were reviewed by a single board-certified radiologist. Consequently, intraobserver and interobserver reliability were not assessed. Although standardized radiological criteria were applied, the possibility of observer-related variability cannot be excluded.

In summary, while flexible fiberoptic endoscopy provides valuable functional and mucosal assessment, its ability to detect AMO is severely compromised in the presence of maxillary sinus opacification and nasal polyps. In patients free of these obstructing pathologies, the FFE detection rate rises to over 50%, a clinically meaningful rate. CT scanning may be particularly useful for pre-operative identification of AMO.

## 5 Conclusion

In this retrospective cohort of patients with CT-documented accessory maxillary ostium, flexible fiberoptic endoscopy visualized the ostium in only 26% of cases overall, indicating limited endoscopic visualization of CT-confirmed AMO. Critically, endoscopic detection was entirely confined to patients without maxillary sinus opacification or nasal polyps, in whom the detection rate reached 51.4%. In contrast, no AMO was endoscopically visualized in any patient with CT-detected opacification or polyps, suggesting that these pathological findings completely obstruct endoscopic visualization of AMO. While FFE remains a valuable, minimally invasive tool for assessing mucosal status and functional drainage within the middle meatus, CT appears more suitable for anatomical identification and characterization of AMO within the limitations of this retrospective design, particularly in patients with concurrent sinus pathology and in preoperative evaluation for functional endoscopic sinus surgery.

## Data Availability

The original contributions presented in the study are included in the article/Supplementary Material, further inquiries can be directed to the corresponding author/s.
